# Can Burnout Be Linked to Suicide Among Healthcare Professionals? A Cross-Sectional Study

**DOI:** 10.3390/nursrep16080276

**Published:** 2026-08-06

**Authors:** Alexandru Ungurianu, Virginia Marina

**Affiliations:** 1 Doctoral School of Biomedical Sciences, “Dunărea de Jos” University of Galati, 47 Str. Domnească, 800146 Galati, Romania; alexungurianumm@gmail.com; 2 Medical Department of Occupational Health, Faculty of Medicine and Pharmacy, Dunărea de Jos” University, 47 Str. Domnească, 800201 Galati, Romania

**Keywords:** burnout, suicidality, healthcare professionals, mental health, occupational health

## Abstract

**Background**: Burnout, defined by emotional exhaustion, depersonalization, and reduced personal accomplishment, is a major occupational health concern among healthcare professionals. Burnout has been linked to depression, anxiety, and medical errors, but its association with suicidality in healthcare settings remains insufficiently explored. **Objective**: To investigate the association between burnout dimensions and suicidality among healthcare professionals, and to identify occupational and demographic factors influencing this relationship. **Methods**: A cross-sectional study was conducted among 195 healthcare professionals in Romania, recruited through voluntary participation in an anonymized online survey. Burnout was assessed using an adapted tool inspired by the Maslach Burnout Inventory, while suicidality outcomes included suicidal ideation, self-harm, and suicide attempts. Descriptive statistics were calculated for demographic and professional variables. Pearson correlations and multivariable linear regression analyses, adjusted for age, professional experience, and shift work, were performed to evaluate associations between burnout dimensions and suicidality. **Results**: Emotional exhaustion (r = 0.42, *p* < 0.001) and depersonalization (r = 0.37, *p* < 0.001) were positively correlated with suicidality, while reduced personal accomplishment was negatively correlated (r = −0.29, *p* = 0.002). In multivariable regression, emotional exhaustion remained the only statistically significant independent predictor of suicidality after adjustment for demographic and occupational characteristics. Younger age and rotating/night-shift work were associated with higher suicidality in unadjusted analyses but were not independent predictors after multivariable adjustment. Strong correlations between burnout dimensions and physical symptoms highlighted the interplay between psychosocial strain and somatic health. **Conclusions**: Burnout, particularly emotional exhaustion, was independently associated with suicidality among healthcare professionals, while depersonalization demonstrated a significant unadjusted association that did not remain statistically significant after multivariable adjustment. Younger clinicians, shift workers, and those with less experience appear especially vulnerable. These findings emphasize that burnout is a multifactorial construct with both psychological and somatic dimensions, shaped by systemic workplace stressors. These findings support further evaluation of systematic screening, organizational reforms, mentorship programs, and confidential mental health support as potential components of suicide-prevention strategies for healthcare professionals.

## 1. Introduction

Healthcare professionals are routinely exposed to demanding working environments characterized by long working hours, high patient acuity, emotionally challenging clinical encounters, ethical dilemmas, and increasing organizational pressures. Prolonged exposure to these occupational stressors contributes to the development of burnout, a multidimensional syndrome characterized by emotional exhaustion, depersonalization, and reduced personal accomplishment [1,2]. Recognized by the World Health Organization as an occupational phenomenon resulting from chronic workplace stress that has not been successfully managed, burnout has become a major concern for healthcare systems worldwide because of its impact on professionals’ well-being, patient safety, and healthcare quality. Recent studies indicate that burnout prevalence frequently exceeds 50% among physicians and nurses, with the COVID-19 pandemic further amplifying emotional strain, professional fatigue, and workforce shortages [1,2,3].

Of particular concern is its relationship with suicidality. Suicide remains one of the leading causes of death worldwide [3], while physicians and nurses consistently demonstrate higher rates of suicidal ideation, suicide attempts, and completed suicide than the general population [4,5]. Epidemiological studies further indicate that female physicians, younger healthcare professionals, and medical residents represent particularly vulnerable subgroups, with excessive workload, shift work, and organizational stressors acting as important contributing factors [4,5,6,7]. Beyond its occupational consequences, burnout has increasingly been associated with adverse mental health outcomes, including depression, anxiety, sleep disturbances, reduced job satisfaction, medical errors, and impaired quality of care [8].

Several theoretical models provide a conceptual framework for understanding the relationship between burnout and suicidality. According to Joiner’s Interpersonal Theory of Suicide, suicidal desire develops through the interaction of perceived burdensomeness and thwarted belongingness, whereas repeated exposure to stressful or painful experiences may increase an individual’s acquired capability for suicide [7,8,9]. Within this framework, emotional exhaustion may intensify feelings of inadequacy and hopelessness, while depersonalization may contribute to emotional withdrawal and reduced interpersonal connectedness. Together, these dimensions may increase psychological vulnerability and facilitate the emergence of suicidal thoughts and behaviors.

Growing empirical evidence supports this conceptual relationship. Recent systematic reviews and meta-analyses have demonstrated that burnout among healthcare professionals is associated with poorer psychological well-being, higher rates of depression and anxiety, and increased suicide risk [1,2,5]. Macaron et al. [1] reported particularly high burnout levels among frontline physicians during the COVID-19 pandemic, whereas Johnson et al. [2] identified excessive workload, insufficient institutional support, and poor work-life balance as major determinants of burnout across healthcare settings. Similarly, Peterson et al. [5] identified healthcare professionals as one of the occupational groups with the highest suicide risk based on national surveillance data. Collectively, these studies suggest that burnout represents not only an occupational phenomenon but also an important public health concern requiring early recognition and intervention.

Despite these advances, several important knowledge gaps remain. Most published studies have evaluated burnout as a single global construct rather than examining the independent contribution of its individual dimensions to suicidality. Furthermore, although numerous studies have been conducted in North America, Western Europe, and Asia, evidence from Eastern European healthcare systems remains comparatively limited. Existing studies have also rarely evaluated the simultaneous influence of demographic and occupational characteristics, such as age, professional experience, and shift work, on the relationship between burnout and suicidality. A better understanding of these multidimensional relationships may facilitate the development of more targeted occupational health interventions and suicide prevention strategies for healthcare professionals [10,11,12,13,14,15,16,17,18].

Therefore, the present cross-sectional study aimed to investigate the association between burnout dimensions and suicidality among Romanian healthcare professionals. Specifically, we examined whether emotional exhaustion, depersonalization, and reduced personal accomplishment were independently associated with suicidality while also evaluating the influence of selected demographic and occupational characteristics. We hypothesized that emotional exhaustion and depersonalization would demonstrate the strongest associations with suicidality, whereas reduced personal accomplishment would show a weaker relationship after adjustment for relevant occupational factors.

Unlike previous studies that have primarily evaluated burnout as a global construct, the present study examined the independent contribution of the three classical burnout dimensions while simultaneously considering physical symptoms and occupational characteristics in a Romanian healthcare population. By providing evidence from an underrepresented Eastern European setting, this study contributes additional data to the growing international literature on burnout and suicidality among healthcare professionals [19,20].

## 2. Materials and Methods

### 2.1. Study Design

This cross-sectional observational study was conducted to investigate the association between burnout dimensions and suicidality among healthcare professionals. Data were collected using an anonymous, self-administered online questionnaire designed specifically for this research. Participation was voluntary, and no financial or material incentives were offered. The survey was available during the study period and could be completed only after participants had provided informed consent electronically. The questionnaire was designed to ensure respondent anonymity, and no personally identifiable information was collected.

As this was an exploratory cross-sectional study employing voluntary convenience sampling, no formal a priori sample size calculation was undertaken before participant recruitment. Nevertheless, to evaluate whether the final sample was adequate for the planned multivariable regression analyses, a post hoc sample size justification was performed. Assuming a medium effect size (f^2^ = 0.15), a significance level (α) of 0.05, statistical power (1 − β) of 0.80, and up to ten independent predictors, the minimum recommended sample size ranged between 118 and 140 participants. Consequently, the final sample of 195 healthcare professionals exceeded this threshold and was considered adequate for detecting moderate associations between burnout dimensions and suicidality.

The sample size estimate was based on the requirements for a fixed-effects multiple linear regression model with up to ten predictors.

### 2.2. Participants

The study included 195 healthcare professionals actively involved in clinical practice, including physicians, nurses, and allied healthcare professionals working in hospitals and outpatient healthcare facilities in Galați County, Romania.

Participants were recruited between 15 January and 20 February 2026, using a voluntary convenience sampling strategy. The questionnaire was disseminated electronically through professional communication channels, including institutional mailing lists and professional social media groups dedicated to healthcare personnel. Participation was entirely voluntary, and no financial or material incentives were offered.

Before accessing the questionnaire, all potential participants received an electronic participant information sheet describing the objectives of the study, the anonymous nature of data collection, the estimated completion time, and their right to withdraw at any time before submitting the questionnaire. Electronic informed consent was obtained from all participants prior to accessing the survey.

Eligible participants were healthcare professionals aged 18 years or older who were actively engaged in clinical practice and voluntarily agreed to participate. Healthcare workers performing exclusively administrative duties, as well as questionnaires with incomplete responses for the primary study variables, were excluded from the final analysis.

Demographic and occupational variables collected included age, sex, marital status, educational attainment, years of professional experience, body mass index (BMI), professional category, and work schedule characteristics (day shifts versus rotating/night shifts).

To minimize duplicate participation, respondents were instructed to complete the questionnaire only once. No personal identifiable information, IP addresses, email addresses, or other identifying information was collected, thereby ensuring complete anonymity throughout the study.

Because the questionnaire link was disseminated through several independent professional communication channels, the exact number of healthcare professionals who received the invitation could not be determined. Consequently, a response rate could not be calculated.

A total of 195 questionnaire records were available in the final dataset. All 195 records contained responses for the primary burnout and suicidality variables and were retained for the principal descriptive and correlation analyses. Because the survey was anonymous and disseminated through multiple channels, the number of individuals who opened the survey but did not submit it could not be determined.

### 2.3. Study Instrument

Data was collected using a structured anonymous online questionnaire comprising six sections: (1) demographic and occupational characteristics, (2) burnout, (3) stress-related physical symptoms, (4) suicidality, (5) depression, and (6) anxiety.

Burnout was assessed using an original questionnaire specifically developed for the present study based on the theoretical framework of the Maslach Burnout Inventory (MBI) [21]. Rather than administering the copyrighted MBI, the questionnaire was designed to reflect the three theoretical dimensions proposed by Maslach and Jackson, Emotional Exhaustion, Depersonalization, and Personal Accomplishment, using original items adapted to the Romanian healthcare context. This approach was selected because permission to reproduce the proprietary MBI items was not available. Consequently, original items were developed to preserve the theoretical constructs of the three burnout dimensions while adapting their wording to reflect occupational experiences encountered by Romanian healthcare professionals, without reproducing the copyrighted wording of the original MBI. The purpose of this adaptation was to enable an exploratory assessment of burnout while respecting copyright restrictions associated with the original instrument.

The burnout questionnaire consisted of 27 items evaluating Emotional Exhaustion, Depersonalization, Personal Accomplishment, and stress-related physical symptoms. Participants rated each item using a seven-point Likert scale ranging from 1 (“Never”) to 7 (“Always”), with higher scores indicating greater symptom frequency. Emotional Exhaustion was calculated from nine items, Depersonalization from five items, Personal Accomplishment from eight items, and Physical Symptoms from five items. Domain scores were calculated by summing the relevant item responses. Personal Accomplishment was interpreted separately from Emotional Exhaustion and Depersonalization, with lower accomplishment reflecting a less favorable outcome. The complete item allocation and scoring procedure are provided in Supplementary Table S1.

Suicidality was assessed using four items derived from the principal domains of the Suicidal Behaviors Questionnaire-Revised (SBQ-R): (1) lifetime suicidal ideation and/or suicide planning, (2) frequency of suicidal ideation during the previous 12 months, (3) communication of suicidal intent to others, and (4) perceived likelihood of a future suicide attempt. Responses were coded ordinally according to increasing severity. For the first item, “Never,” “It was just a brief passing thought,” and “I have had a plan at least once but did not attempt suicide” were scored 1, 2, and 3, respectively. For the second item, “Never,” “Rarely (once),” “Sometimes (2–3 times),” and “Often” were scored 1, 2, 3, and 4, respectively. For the third item, “No,” “Yes, at one time, but I really did not want to die,” and “Yes, at one time, and I really wanted to die” were scored 1, 2, and 3, respectively. For the fourth item, “Never,” “No chance at all,” and “Rather unlikely” were scored 0, 1, and 2, respectively. The four scores were summed to produce an exploratory suicidality composite ranging theoretically from 3 to 12; observed scores in the present sample ranged from 3 to 11, with higher scores indicating greater suicidal vulnerability.

The four-item composite demonstrated high internal consistency in the present sample (Cronbach’s α = 0.903). Nevertheless, this coefficient should be interpreted cautiously because of the restricted response range and strong inter-item dependence. Because the wording and available response categories differed from the original SBQ-R, established SBQ-R clinical cut-off values were not applied. The composite was analyzed as an exploratory continuous outcome and should not be interpreted as a clinical diagnosis or as equivalent to the validated SBQ-R score. Further evaluation of its factor structure, convergent validity, criterion validity, and test–retest reliability is required. The original SBQ-R has demonstrated good psychometric properties in clinical and non-clinical populations [22].

Depressive and anxiety symptoms were explored using two study-specific symptom-frequency indicators informed by the content and response framework of the PHQ-9 and GAD-7. Participants were presented with the symptom domains covered by each instrument and provided a single overall frequency response for each domain using a four-point scale: 0 (“Not at all”), 1 (“Several days”), 2 (“More than half the days”), or 3 (“Nearly every day”). Because individual responses to all nine PHQ-9 items and all seven GAD-7 items were not recorded separately, validated PHQ-9 and GAD-7 total scores could not be calculated. These variables should therefore not be interpreted as complete PHQ-9 or GAD-7 measurements and were not used for clinical classification or included in the final multivariable regression model. The original PHQ-9 and GAD-7 instruments and their psychometric properties are described in their respective validation studies [23,24,25].

The psychometric properties of the adapted burnout questionnaire were evaluated by assessing internal consistency using Cronbach’s alpha coefficients. Emotional Exhaustion demonstrated excellent internal consistency (α = 0.895), Depersonalization showed good reliability (α = 0.808), Physical Symptoms also demonstrated good reliability (α = 0.861), whereas Personal Accomplishment demonstrated moderate internal consistency (α = 0.576). The overall burnout questionnaire demonstrated excellent internal consistency (Cronbach’s α = 0.902), supporting its use as an exploratory research instrument. Nevertheless, because the questionnaire was specifically developed for the present study based on the theoretical framework of the Maslach Burnout Inventory rather than using the original validated instrument, additional psychometric evaluation, including construct validity, convergent validity, confirmatory factor analysis, and test- retest reliability, is recommended before broader research or clinical application.

Missing data were minimal, and no imputation was performed. Descriptive and bivariate analyses used all available observations for the variables involved. The final multivariable regression model was based on 190 analyzable cases after complete-case and data-quality screening. Five records were not included because one or more variables required for the final model were unavailable or not suitable for analysis.

Responses to the four suicidality items were coded according to the response categories used in the study questionnaire and summed to produce a composite suicidality score ranging from 0 to 3, with higher scores indicating greater suicidal vulnerability. Because the wording and/or response structure differed from the original SBQ-R, established SBQ-R cut-off values were not applied. The adapted suicidality measure was treated as an exploratory continuous outcome, and its results should not be interpreted as a clinical diagnosis or as equivalent to the validated SBQ-R score.

### 2.4. Statistical Analysis

Statistical analyses were performed using IBM SPSS Statistics for Windows, Version 29.0 (IBM Corp., Armonk, NY, USA).

Continuous variables were summarized as means, standard deviations (SD), medians, minimum values, and maximum values, whereas categorical variables were presented as absolute frequencies and percentages.

The distribution of continuous variables was evaluated using the Kolmogorov–Smirnov test with Lilliefors correction. Variables demonstrating approximately normal distributions were analyzed using parametric statistical methods, whereas non-normally distributed variables were interpreted with caution, considering the exploratory nature of the analyses.

Associations between categorical variables were examined using Pearson’s chi-square test. Fisher’s exact test was applied whenever expected cell frequencies did not satisfy the assumptions required for the chi-square test.

Comparisons between two independent groups were performed using Student’s independent-samples *t*-test. Comparisons involving more than two groups were conducted using one-way analysis of variance (ANOVA), followed by Bonferroni post hoc testing when appropriate.

Relationships between burnout dimensions, physical symptoms, demographic variables, and suicidality were assessed using Pearson correlation coefficients.

To identify independent predictors of suicidality, multivariable linear regression analyses were performed. The dependent variable was the suicidality score, while burnout dimensions (Emotional Exhaustion, Depersonalization, and Personal Accomplishment), together with selected demographic and occupational variables (age, years of professional experience, and shift work.), were included as independent variables. Depressive and anxiety symptom-frequency indicators were collected as part of the study questionnaire. However, because individual PHQ-9 and GAD-7 item responses were not recorded separately, validated total scores could not be calculated. These exploratory indicators were therefore not entered into the final multivariable regression model. Prior to model estimation, the assumptions of linear regression were evaluated, including linearity, independence of observations, homoscedasticity, normality of residuals, and multicollinearity. Multicollinearity was assessed using tolerance values and the Variance Inflation Factor (VIF), and no evidence of problematic multicollinearity was identified. Regression results are presented as unstandardized regression coefficients (B), standard errors (SE), standardized coefficients (β), 95% confidence intervals (95% CI), t statistics, and corresponding *p*-values. Overall model performance was evaluated using the coefficient of determination (R^2^), adjusted R^2^, and the F statistic.

A two-sided *p*-value < 0.05 was considered statistically significant for all analyses.

Because multiple hypothesis tests were performed throughout the correlation and regression analyses, *p*-values were additionally adjusted using the Benjamini–Hochberg false discovery rate (FDR) procedure. Bonferroni correction was retained for post hoc pairwise comparisons following one-way ANOVA.

Although several variables showed departures from normality according to the Kolmogorov–Smirnov test, the relatively large sample size (number: 95) allowed the use of parametric analyses based on the robustness of these methods under the Central Limit Theorem. Consequently, Pearson correlation coefficients and multivariable linear regression were considered appropriate for the exploratory analyses performed in the present study.

All statistically significant associations remained significant after Benjamini–Hochberg false discovery rate adjustment.

### 2.5. Ethics

The study was conducted in accordance with the ethical principles of the Declaration of Helsinki.

Ethical approval was obtained from the Ethics Committee of the Clinical Emergency Hospital, Galați, Romania (approval number 10889/18 October 2025).

Participation was voluntary, and all participants provided informed electronic consent before accessing the questionnaire. The survey was completed anonymously, and no personally identifiable information was collected or stored. Participants were informed about the objectives of the study, the confidential handling of the data, and their right to discontinue participation at any time without consequences.

## 3. Results

### 3.1. Participant Characteristics

A total of 195 submitted questionnaires met the eligibility criteria and were included in the main analyses. The demographic and occupational characteristics of the study population are summarized in Table 1. Participants had a mean age of 47.6 ± 11.7 years, and the majority were female (64.1%). Most respondents were married (65.6%) and held at least a bachelor’s degree (70.8%), while 17.4% had obtained a doctoral degree. The mean duration of professional experience was 20.8 ± 13.0 years. Body mass index (BMI) data were available for 193 participants, with a mean value of 26.5 ± 4.6 kg/m^2^, indicating that the cohort was, on average, overweight. Approximately 70% of participants reported more than 10 years of professional experience, and a substantial proportion worked rotating or night shifts.

After harmonization of the free-text professional titles, 139 participants (71.3%) were classified as physicians or dentists, 44 (22.6%) as nursing or midwifery personnel, and 12 (6.2%) as allied or other healthcare-related professionals.

### 3.2. Internal Consistency of the Questionnaire

The internal consistency of the adapted questionnaire was evaluated using Cronbach’s alpha coefficient. The overall questionnaire demonstrated excellent internal consistency (Cronbach’s α = 0.902).

Among the individual domains, Emotional Exhaustion demonstrated good-to-excellent reliability (α = 0.895), Depersonalization showed good internal consistency (α = 0.808), and the Physical Symptoms domain also demonstrated good reliability (α = 0.861). The Personal Accomplishment domain showed lower internal consistency (α = 0.576), indicating greater heterogeneity among the items included within this construct (Table 2).

Because the questionnaire was specifically developed for this exploratory study, construct validity was inferred from its theoretical alignment with the three Maslach burnout dimensions rather than from confirmatory factor analysis. Formal validation of the instrument, including exploratory and confirmatory factor analyses, should be performed in future studies.

### 3.3. Burnout Dimensions and Physical Symptoms

Burnout levels across the three assessed dimensions were examined descriptively. High emotional exhaustion was observed in 60.5% of participants, representing the most prevalent burnout dimension. High depersonalization was reported by 51.3% of respondents, whereas only 6.7% demonstrated high levels of reduced personal accomplishment. Most participants (69.7%) maintained preserved professional accomplishment.

Participants also reported several physical symptoms commonly associated with occupational stress. Musculoskeletal pain represented the most frequently reported complaint, followed by headaches and gastrointestinal symptoms, whereas cardiovascular complaints were less frequently reported. Overall, the physical symptom score suggested a moderate burden of stress-related somatic manifestations.

### 3.4. Correlations Between Burnout and Suicidality

Pearson correlation analyses demonstrated significant associations between burnout dimensions and suicidality (Table 3). Emotional exhaustion showed the strongest positive correlation with suicidality (r = 0.42, *p* < 0.001), followed by depersonalization (r = 0.37, *p* < 0.001). Reduced personal accomplishment was negatively correlated with suicidality (r = −0.29, *p* = 0.002). Burnout was also strongly associated with physical symptom burden (r = 0.81, *p* < 0.001), while a weaker positive association was observed with rotating or on-call shift work (r = 0.26, *p* < 0.05). Overall, higher burnout severity was associated with greater suicidality and increased physical symptom burden.

The reported *p*-values are unadjusted. All statistically significant findings remained significant after Benjamini–Hochberg FDR correction.

### 3.5. Multivariable Regression Analysis

A multivariable linear regression analysis was conducted to identify independent predictors of suicidality after adjustment for age, professional experience and work schedule (Table 4). Burnout dimensions (Emotional Exhaustion, Depersonalization, and Personal Accomplishment) were entered simultaneously into the model together with these demographic and occupational covariates.

The overall regression model was statistically significant (*F*(6, 183) = 13.19, *p* < 0.001), explaining 30.2% of the variance in suicidality scores (R^2^ = 0.302; adjusted R^2^ = 0.279). Emotional Exhaustion emerged as the only statistically significant independent predictor of suicidality (B = 0.108, SE = 0.017, standardized β = 0.546, 95% CI: 0.074–0.143, *p* < 0.001). In contrast, Depersonalization, Personal Accomplishment, age, professional experience, and on-call shift work were not independently associated with suicidality after multivariable adjustment. Detailed regression coefficients, confidence intervals, and multicollinearity diagnostics are presented in Table 4.

### 3.6. Subgroup Analyses

Subgroup analyses evaluated the influence of demographic and occupational variables on burnout and suicidality.

Healthcare professionals younger than 35 years reported significantly higher suicidality scores than older participants (*p* < 0.05). Among female participants, increasing age was significantly associated with higher emotional exhaustion (*p* = 0.013), whereas among male participants, age was positively associated with depersonalization (*p* = 0.042).

Participants working rotating or night shifts generally reported higher burnout and suicidality scores than those working exclusively daytime schedules, although some subgroup comparisons did not consistently reach statistical significance. Higher BMI was moderately associated with emotional exhaustion and physical symptom burden (*p* < 0.05).

## 4. Discussion

### 4.1. Principal Findings

The present study investigated the association between burnout dimensions and suicidality among Romanian healthcare professionals. Emotional exhaustion emerged as the only statistically significant independent predictor of suicidality after adjustment for demographic and occupational characteristics. Although depersonalization and reduced personal accomplishment were associated with suicidality in the unadjusted analyses, these associations did not remain statistically significant after multivariable adjustment. Burnout was also significantly associated with physical symptoms and occupational factors, particularly rotating or night-shift work.

Importantly, the adapted questionnaire demonstrated excellent overall internal consistency (Cronbach’s α = 0.902), with good-to-excellent reliability for the Emotional Exhaustion (α = 0.895), Depersonalization (α = 0.808), and Physical Symptoms (α = 0.861) domains. The Personal Accomplishment domain demonstrated lower internal consistency (α = 0.576), suggesting greater heterogeneity among its constituent items and indicating that findings involving this dimension should be interpreted cautiously.

### 4.2. Interpretation of Findings

Our findings support the growing body of evidence indicating that burnout is closely associated with adverse mental health outcomes among healthcare professionals. Emotional exhaustion demonstrated the strongest association with suicidality, suggesting that prolonged emotional depletion may be an important psychological pathway through which occupational stress is related to suicidal thoughts. Although depersonalization demonstrated a significant positive correlation with suicidality, its association was attenuated after adjustment for emotional exhaustion and demographic variables, suggesting that emotional exhaustion may account for a substantial proportion of the observed relationship between burnout and suicidality.

These findings are consistent with Joiner’s Interpersonal Theory of Suicide, which proposes that perceived burdensomeness and thwarted belongingness contribute to suicidal desire. Emotional exhaustion may increase feelings of inadequacy and hopelessness, whereas depersonalization may reduce interpersonal connectedness, thereby creating conditions that facilitate suicidal ideation. Similarly, the Job Demands–Resources model and Conservation of Resources theory provide complementary explanations by emphasizing how persistent occupational demands combined with insufficient recovery progressively deplete emotional and cognitive resources, ultimately increasing vulnerability to psychological distress [8,9,10,11,12,13,14].

### 4.3. Comparison with Previous Literature

The present findings are consistent with previous international studies reporting high levels of burnout among healthcare professionals and its association with poorer psychological well-being [1,2]. The prevalence of high emotional exhaustion (60.5%) and depersonalization (51.3%) observed in our cohort closely mirrors findings reported among physicians and nurses worldwide, particularly following the COVID-19 pandemic [1,2].

Our results also support previous reports demonstrating elevated suicide risk among healthcare professionals compared with the general population [4,5,6,7,8,9]. Similar to earlier studies, younger healthcare professionals and individuals working rotating or night shifts appeared particularly vulnerable. By providing evidence from Romania, our study expands the relatively limited body of literature from Eastern European healthcare systems and contributes additional regional evidence to this growing field.

The strong association between burnout and physical symptoms (r = 0.81) is consistent with the multidimensional nature of burnout. Musculoskeletal pain, headaches, gastrointestinal complaints, and cardiovascular symptoms may reflect somatic manifestations associated with chronic occupational stress and may further contribute to psychological burden.

The particularly strong correlation observed between burnout and physical symptoms may reflect the multidimensional nature of chronic occupational stress, in which psychological distress frequently manifests through somatic complaints. Nevertheless, because both constructs were assessed using self-reported measures collected at the same time point, the possibility of shared method variance and partial conceptual overlap cannot be excluded. Therefore, this finding should be interpreted cautiously until confirmed in longitudinal studies using objective clinical measures.

### 4.4. Clinical Implications

The present findings may have implications for occupational health practice. Burnout, particularly emotional exhaustion, may represent a clinically relevant marker of psychological vulnerability among healthcare professionals. Healthcare organizations may consider evaluating strategies aimed at reducing excessive workload, improving staffing, optimizing shift schedules, strengthening institutional support, and ensuring confidential access to psychological services. Younger professionals and those working rotating or night shifts may warrant particular attention in future prospective prevention studies.

### 4.5. Strengths and Limitations

This study has several strengths. It evaluated multiple dimensions of burnout simultaneously while also incorporating physical symptoms and occupational characteristics into the analysis. Furthermore, the adapted questionnaire demonstrated excellent overall internal consistency and good reliability for most assessed domains, supporting its suitability for exploratory research.

Several limitations should nevertheless be acknowledged. First, the cross-sectional design precludes conclusions regarding causality. Second, the use of self-reported questionnaires introduces the possibility of recall and social desirability bias. Third, participants were recruited using convenience sampling from a single Romanian region, which may limit the generalizability of the findings.

In addition, although the questionnaire demonstrated excellent overall reliability, the Personal Accomplishment domain showed lower internal consistency than the remaining domains. This finding suggests greater heterogeneity among the included items and indicates that results involving this dimension should be interpreted cautiously. Further validation of the adapted questionnaire in larger and more diverse populations is warranted.

Additionally, several potentially important confounding variables were not fully evaluated or included in the multivariable analyses, including previous psychiatric disorders, psychotropic medication use, sleep quality, alcohol consumption, weekly working hours, workload, medical specialty, organizational support, and workplace climate. Although depressive and anxiety symptoms were explored using indicators informed by the PHQ-9 and GAD-7 frameworks, individual item-level responses were not recorded separately and validated total scores could not be calculated. Consequently, depression and anxiety could not be included as validated covariates in the adjusted regression model, and residual confounding by mental health status cannot be excluded. Future studies should administer and score the complete PHQ-9 and GAD-7 instruments and include these variables in multivariable analyses. Because anonymous convenience sampling was employed, the response rate could not be determined, and volunteer bias cannot be excluded. Participants with a greater interest in mental health or burnout may have been more likely to complete the questionnaire, potentially limiting the representativeness of the sample.

### 4.6. Future Research

Future longitudinal and multicenter studies are needed to clarify the temporal relationship between burnout and suicidality and to evaluate whether interventions targeting burnout reduce suicide risk among healthcare professionals. Further psychometric validation of the adapted questionnaire should also be undertaken, including confirmatory factor analysis and assessment of construct validity. The integration of biological markers of chronic stress together with machine learning approaches may improve the early identification of healthcare professionals at increased risk of burnout and suicidality.

### 4.7. Conclusions

Burnout, particularly emotional exhaustion, was independently associated with suicidality, while depersonalization demonstrated a significant unadjusted association that did not remain statistically significant after multivariable adjustment. Younger professionals, individuals with fewer years of professional experience, and those working rotating or night shifts appeared particularly vulnerable. The adapted questionnaire demonstrated excellent overall reliability, supporting its application in exploratory occupational health research.

These findings suggest that burnout, particularly emotional exhaustion, may represent an important marker of suicide risk among healthcare professionals. Organizational strategies aimed at reducing occupational stress and strengthening psychological support may contribute to improving healthcare professionals’ well-being and should be evaluated in prospective studies.

## Figures and Tables

**Table 1:** Demographic and professional characteristics of the participants.

Characteristic	n (%) or Mean ± SD
Age, years	47.6 ± 11.7
Age group, n (%)	
25–30 years	15 (7.7%)
30–35 years	20 (10.3%)
35–45 years	46 (23.6%)
45–55 years	65 (33.3%)
55–65 years	34 (17.4%)
>65 years	15 (7.7%)
Sex, n (%)	
Female	125 (64.1%)
Male	70 (35.9%)
Marital status, n (%)	
Married	128 (65.6%)
Single	33 (16.9%)
Divorced	30 (15.4%)
Widowed	4 (2.1%)
Educational level, n (%)	
Post-secondary education	23 (11.8%)
Bachelor’s degree	138 (70.8%)
Doctorate	34 (17.4%)
Professional experience, years	20.8 ± 13.0
Professional category, n (%)	
Physicians and dentists	139 (71.3%)
Nursing and midwifery personnel	44 (22.6%)
Allied/other healthcare-related roles	12 (6.2%)
Body mass index, kg/m^2^	26.5 ± 4.6

**Table 2:** Internal consistency of the questionnaire domains.

Domain	Number of Items	Cronbach’s α	Interpretation
Emotional Exhaustion	9	0.895	Good to excellent
Depersonalization	5	0.808	Good
Personal Accomplishment	8	0.576	Lower internal consistency
Physical Symptoms	5	0.861	Good
Overall questionnaire	27	0.902	Excellent

**Table 3:** Correlations between burnout dimensions, suicidality, and occupational factors.

Variable	r	*p*-Value
Emotional Exhaustion → Suicidality	0.42	<0.001
Depersonalization → Suicidality	0.37	<0.001
Reduced Personal Accomplishment → Suicidality	–0.29	0.002
Burnout → Physical symptoms	0.81	<0.001
Burnout → On-call shifts	0.26	<0.05

**Table 4:** Multivariable linear regression analysis of factors associated with suicidality among healthcare professionals.

Predictor	B	SE	Standardized β	95% CI for B	t	*p*-Value	VIF
Emotional Exhaustion	0.108	0.017	0.546	0.074 to 0.143	6.224	<0.001	2.020
Depersonalization	−0.021	0.038	−0.051	−0.096 to 0.053	−0.566	0.572	2.122
Personal Accomplishment	0.013	0.024	0.034	−0.034 to 0.060	0.537	0.592	1.037
Age	−0.029	0.031	−0.138	−0.090 to 0.032	−0.929	0.354	5.782
Professional experience	−0.007	0.028	−0.038	−0.062 to 0.048	−0.256	0.798	5.905
On-call shift work	−0.130	0.312	−0.027	−0.745 to 0.486	−0.416	0.678	1.066
Constant	2.990	1.404	-	0.219 to 5.760	2.129	0.035	-

Model statistics: *N* = 190; R^2^ = 0.302; adjusted R^2^ = 0.279; *F*(6, 183) = 13.188; *p* < 0.001; Durbin–Watson = 2.095. Abbreviations: B, unstandardized regression coefficient; SE, standard error; β, standardized regression coefficient; CI, confidence interval; VIF, variance inflation factor.

## Data Availability

The dataset supporting the findings of this study is available upon request from the corresponding author.

## Supplementary Materials

The following supporting information can be downloaded at https://www.mdpi.com/article/10.3390/nursrep16080276/s1, Table S1: Item allocation and scoring procedure for the study questionnaire; File S1: STROBE Statement—Checklist for Cross-Sectional Studies.

## Public Involvement Statement

No public involvement in any aspect of this research.

## Guidelines and Standards Statement

This manuscript was drafted in accordance with the Strengthening the Reporting of Observational Studies in Epidemiology (STROBE) Statement for reporting cross-sectional observational studies (von Elm E, Altman DG, Egger M, et al. The Strengthening the Reporting of Observational Studies in Epidemiology (STROBE) Statement: Guidelines for Reporting Observational Studies. PLoS Med. 2007; 4(10): e296. https://doi.org/10.1371/journal.pmed.0040296).
