# Supplementary material for: Can Burnout Be Linked to Suicide Among Healthcare Professionals? A Cross-Sectional Study
Source: Nurs Rep. 2026 Aug 6;16(8):276. doi: 10.3390/nursrep16080276 (PMC13516551; doi:10.3390/nursrep16080276)
Supplement: Supplementary file 1 [file nursrep-16-00276-s001.zip › nursrep-4431702-supplementary.pdf]

Supplementary Table S1. Item allocation and scoring procedure for the study questionnaire

Domain	Item No.	Item	Response scale	Scoring	Domain score
Emotional Exhaustion	1	I feel emotionally drained because of my work.	1 = Never to 7 = Always	Directly scored	Sum of Items 1, 2, 3, 6, 8, 13, 14, 16, and 20; range 9–63
Emotional Exhaustion	2	I feel worn out at the end of a workday.	1–7	Directly scored	—
Emotional Exhaustion	3	I feel tired when I wake up in the morning and must face another workday.	1–7	Directly scored	—
Emotional Exhaustion	6	Working all day among patients is a real discomfort for me.	1–7	Directly scored	—
Emotional Exhaustion	8	I feel professionally exhausted because of my work.	1–7	Directly scored	—
Emotional Exhaustion	13	I do not feel confident about performing my job efficiently.	1–7	Directly scored	—
Emotional Exhaustion	14	I think I work too much.	1–7	Directly scored	—
Emotional Exhaustion	16	Working directly with people creates too much tension for me.	1–7	Directly scored	—
Emotional Exhaustion	20	I feel like I cannot do this job anymore.	1–7	Directly scored	—
Depersonalization	5	I feel that I treat certain patients as if they were objects.	1–7	Directly scored	Sum of Items 5, 10, 11, 15, and 22; range 5–35
Depersonalization	10	I feel that my level of empathy has significantly reduced since starting this job.	1–7	Directly scored	—
Depersonalization	11	I fear that this job will harden me emotionally.	1–7	Directly scored	—
Depersonalization	15	I do not really care about what happens to some patients.	1–7	Directly scored	—
Depersonalization	22	I feel that my patients blame me for their problems.	1–7	Directly scored	—
Personal Accomplishment	4	I imagine that even patients think I am tired and that I will not perform a proper consultation.	1–7	Verify whether directly or reverse scored	Sum of Items 4, 7, 9, 12, 17, 18, 19, and 21; range 8–56
Personal Accomplishment	7	I am effective in solving problems related to my work and my patients.	1–7	Verify scoring direction	—
Personal Accomplishment	9	I feel that I make a positive contribution to the lives of those around me through my work.	1–7	Verify scoring direction	—
Personal Accomplishment	12	I feel full of energy.	1–7	Verify scoring direction	—
Personal Accomplishment	17	During consultations, I can offer my patients a sense of comfort and relaxation.	1–7	Verify scoring direction	—
Personal Accomplishment	18	I feel happy after working with my patients.	1–7	Verify scoring direction	—
Personal Accomplishment	19	I have achieved many valuable things in my professional work.	1–7	Verify scoring direction	—
Personal Accomplishment	21	In my professional activity, I approach emotional issues calmly.	1–7	Verify scoring direction	—
Physical Symptoms	25	I experience palpitations or chest pain.	1–7	Directly scored	Sum of Items 25–29; range 5–35
Physical Symptoms	26	I have stomach and/or digestive problems.	1–7	Directly scored	—
Physical Symptoms	27	I have headaches.	1–7	Directly scored	—
Physical Symptoms	28	I have muscle pain, for example, in my neck, shoulders, or back.	1–7	Directly scored	—
Physical Symptoms	29	I often get sick.	1–7	Directly scored	—

Suicidality

Construct	Item	Response coding	Score range
Suicidality Item 1	Have you ever thought about or attempted to kill yourself?	Never = 1; brief passing thought = 2; plan without attempt = 3	1–3
Suicidality Item 2	During the past 12 months, how often have you thought about killing yourself?	Never = 1; rarely = 2; sometimes = 3; often = 4	1–4
Suicidality Item 3	Have you ever told someone that you were going to commit suicide, or that you might do it?	No = 1; yes, without intent to die = 2; yes, with intent to die = 3	1–3
Suicidality Item 4	How likely is it that you will attempt suicide someday?	Never = 0; no chance at all = 1; rather unlikely = 2	0–2
Composite suicidality score	Sum of the four items	Higher scores indicate greater suicidal vulnerability	3–12

The suicidality items were adapted from the domains of the SBQ-R. Because wording and response categories differed from the validated SBQ-R, established clinical cut-offs were not applied, and the composite was treated as an exploratory continuous outcome.

Supplementary File S1. STROBE checklist for cross-sectional studies, including the manuscript page and line number corresponding to each applicable item.

STROBE Statement—Checklist for Cross-Sectional Studies

Manuscript: Can Burnout Be Linked to Suicide among Healthcare Professionals? A Cross-Sectional Study

The page/section references below correspond to the current 19-page manuscript version. Update the page numbers after final typesetting. Items marked “Partial” require the noted revision before submission.

Item	Section	STROBE recommendation	Reported on page/section	Status	Notes / required action
1a	Title and abstract	Indicate the study’s design with a commonly used term in the title or abstract.	Title, p. 1; Abstract, p. 2	Complete	The title identifies the work as a cross-sectional study.
1b	Title and abstract	Provide in the abstract an informative and balanced summary of what was done and what was found.	Abstract, p. 2	Complete	Design, sample, instruments, analyses, principal results, and cautious conclusions are reported.
2	Background/rationale	Explain the scientific background and rationale for the investigation being reported.	Introduction, pp. 3–4	Complete	The occupational, mental-health, and suicide-prevention rationale is described.
3	Objectives	State specific objectives, including any prespecified hypotheses.	Introduction, p. 4	Complete	The study objective and hypotheses concerning the three burnout dimensions are stated.
4	Study design	Present key elements of study design early in the paper.	Section 2.1, p. 4	Complete	The manuscript identifies the study as cross-sectional and observational.
5	Setting	Describe the setting, locations, and relevant dates, including periods of recruitment and data collection.	Section 2.2, p. 5	Partial	Galați County, hospitals/outpatient facilities, and 15 January–20 February 2026 are reported. Add the number and type of participating institutions if available.
6a	Participants	Give eligibility criteria and the sources and methods of selection of participants.	Section 2.2, p. 5	Complete	Eligibility, exclusion criteria, convenience sampling, dissemination channels, consent, and anonymity are described.

6b	Participants	For matched studies, give matching criteria and numbers of exposed and unexposed participants.	Not applicable	N/A	The study was not matched.
7	Variables	Clearly define all outcomes, exposures, predictors, potential confounders, and effect modifiers. Give diagnostic criteria, if applicable.	Sections 2.2–2.4, pp. 5–8	Partial	Burnout domains and covariates are defined. Insert the exact suicidality coding, score range, and statement that no diagnostic cut-off was applied.
8	Data sources/measurement	For each variable of interest, give sources of data and details of methods of assessment. Describe comparability of assessment methods if there is more than one group.	Section 2.3, pp. 5–7; Supplementary Table S1	Partial	Burnout item allocation and reliability are reported. Finalize Supplementary Table S1 and accurately describe the PHQ-9/GAD-7-derived indicators rather than complete validated totals.
9	Bias	Describe any efforts to address potential sources of bias.	Section 2.2, p. 5; Section 4.5, p. 16	Complete	Duplicate prevention, anonymity, volunteer bias, selection bias, recall bias, and social-desirability bias are discussed.
10	Study size	Explain how the study size was arrived at.	Section 2.1, pp. 4–5	Complete	A post hoc fixed-effects multiple-regression justification is provided with effect size, alpha, power, and predictor count.
11	Quantitative variables	Explain how quantitative variables were handled in the analyses. If applicable, describe which groupings were chosen and why.	Sections 2.3–2.4, pp. 6–8; Table 1, p. 9	Partial	Continuous summaries and age categories are reported. Add the rationale or source for burnout severity categories/cut-offs if these categories are retained.
12a	Statistical methods	Describe all statistical methods, including those used to control for confounding.	Section 2.4, pp. 7–8	Complete	Descriptive, chi-square/Fisher, t-test, ANOVA, correlations, multivariable regression, assumptions, and diagnostics are described.
12b	Statistical methods	Describe any methods used to examine subgroups and interactions.	Section 2.4, pp. 7–8; Section 3.6, p. 12	Partial	Subgroup results are reported, but the Methods should explicitly state which subgroup comparisons were prespecified and how they were tested.
12c	Statistical methods	Explain how missing data were addressed.	Missing data subsection, p. 7	Partial	Complete-case analysis and no imputation are reported. Explain why the final regression used 190 rather than 195 cases.
12d	Statistical methods	If applicable, describe analytical methods taking account of sampling strategy.	Section 2.2, p. 5	N/A	No complex survey sampling or weighting was used.
12e	Statistical methods	Describe any sensitivity analyses.	Not reported	Partial	No sensitivity analysis is described. State explicitly that no formal sensitivity analyses were performed, if correct.
13a	Participants	Report numbers of individuals at each stage of the study.	Section 3.1, p. 9	Partial	The final sample of 195 is reported. Add the number of submitted questionnaires and the number excluded before reaching the final sample.
13b	Participants	Give reasons for non-participation at each stage.	Section 2.2, p. 5	Partial	The denominator was unavailable because the anonymous link was distributed through several channels. Report exclusion reasons/counts for submitted questionnaires, if any.
13c	Participants	Consider use of a flow diagram.	Not included	Optional	A simple flow diagram is recommended if received/excluded/analyzed counts can be reconstructed.
14a	Descriptive data	Give characteristics of study participants and information on exposures and potential confounders.	Section 3.1 and Table 1, p. 9	Partial	Demographic characteristics are reported. Add professional-category frequencies (physicians/dentists, nursing/midwifery, allied/other roles).
14b	Descriptive data	Indicate the number of participants with missing data for each variable of interest.	Missing data subsection, p. 7; Section 3.1, p. 9	Partial	BMI and physical-symptom missingness are reported. Add missingness for all variables used in the regression or state that none were missing.
14c	Descriptive data	Summarize follow-up time.	Not applicable	N/A	Cross-sectional study with no follow-up.

15	Outcome data	Report numbers of outcome events or summary measures.	Sections 3.3–3.5, pp. 10–12; Tables 3–4	Partial	Burnout prevalence, correlations, and regression results are reported. Add descriptive statistics for the suicidality composite, including mean, SD, median, and observed range.
16a	Main results	Give unadjusted estimates and, if applicable, confounder-adjusted estimates and their precision. Make clear which confounders were adjusted for and why.	Sections 3.4–3.5, pp. 11–12; Tables 3–4	Complete	Unadjusted correlations and adjusted regression coefficients with 95% CIs are reported.
16b	Main results	Report category boundaries when continuous variables were categorized.	Table 1, p. 9; Section 3.3, p. 10	Partial	Age-group boundaries are shown. Add the thresholds used to classify low/moderate/high burnout if such classifications are retained.
16c	Main results	If relevant, consider translating estimates of relative risk into absolute risk.	Not applicable	N/A	The study used correlations and linear regression rather than relative-risk estimates.
17	Other analyses	Report other analyses performed, such as subgroup analyses, interactions, and sensitivity analyses.	Section 3.6, p. 12	Partial	Subgroup analyses are reported. Clarify whether they were prespecified and whether multiplicity correction covered these comparisons.
18	Key results	Summarize key results with reference to study objectives.	Section 4.1, p. 13	Complete	Principal findings are summarized and aligned with the adjusted model.
19	Limitations	Discuss limitations, taking account of sources of potential bias or imprecision. Discuss direction and magnitude of potential bias.	Section 4.5, p. 16	Complete	Cross-sectional design, self-report, convenience sampling, psychometric limitations, residual confounding, and volunteer bias are discussed.
20	Interpretation	Give a cautious overall interpretation considering objectives, limitations, multiplicity of analyses, results from similar studies, and other relevant evidence.	Sections 4.2–4.7, pp. 13–17	Complete	The language is predominantly associative and the conclusions acknowledge the design limitations.
21	Generalisability	Discuss the generalisability of the study results.	Section 4.5, p. 16	Complete	The single-region convenience sample and limited representativeness are acknowledged.
22	Funding	Give the source of funding and the role of the funders.	Funding statement, p. 17	Complete	The manuscript states that no external funding was received.
